# Dual-Channel NIR Fluorescence Imaging for Precise Delineation of Gastric Tumor Margins

**DOI:** 10.34133/bmr.0275

**Published:** 2025-10-28

**Authors:** Yuanyuan Ji, Kai Bao, Lin Mei, Yuanhao Su, Yongke Wu, Cheng Li, Yongshen Wu, Zhishen Ge, Sangkee Choi, Zhidong Wang, Hak Soo Choi

**Affiliations:** ^1^ Department of Geriatric General Surgery, The Second Affiliated Hospital, Xian Jiaotong University, Xian, China.; ^2^ Scientific Research Center and Precision Medical Institute, The Second Affiliated Hospital, Xian Jiaotong University, Xian, China.; ^3^ Department of Radiology, Gordon Center for Medical Imaging, Massachusetts General Hospital and Harvard Medical School, Boston, MA, USA.; ^4^ MOE Key Laboratory for Nonequilibrium Synthesis and Modulation of Condensed Matter, School of Physics, Xian Jiaotong University, Xian, China.; ^5^ School of Chemistry, Xian Key Laboratory of Sustainable Polymer Materials, Xian Jiaotong University, Xian, China.; ^6^ School of Materials Science, Japan Advanced Institute of Science and Technology, Ishikawa, Japan.

## Abstract

Fluorescence imaging is a promising intraoperative technique for gastric cancer surgery, enabling clear visualization of surgical margins and detection of occult lesions. However, the lack of near-infrared (NIR) fluorescent probes specifically targeting gastric tumors and normal tissues remains a limitation. To address this, we developed a dual-channel imaging strategy using IR-780 (800 nm) for tumor detection and ESS65-Cl (700 nm) for normal gastric tissue identification. We evaluated their specificity in human gastric epithelial (GES-1) and cancer (SGC-7901) cells, confirming selective uptake: ESS65-Cl in normal gastric cells and IR-780 in tumor cells. In subcutaneous and orthotopic xenograft models, dual-channel imaging allowed simultaneous visualization of tumors and surrounding tissues in distinct colors. Pharmacokinetic analysis revealed that ESS65-Cl achieved a stomach signal-to-background ratio of 3.3 by 48 h, while IR-780 exhibited a tumor-to-background ratio of 4.0, demonstrating high targetability. Moreover, biodistribution studies confirmed efficient clearance of both agents. When combined, these fluorophores enabled precise intraoperative differentiation between gastric tissues and tumors. This approach holds substantial potential for improving surgical accuracy in gastric cancer resection, particularly in defining proximal esophageal margins and gastrectomy boundaries. By enhancing real-time tissue discrimination, dual-channel NIR imaging may increase surgical success rates and improve patient outcomes.

## Introduction

Gastric cancer (GC) remains a significant global health concern, with over one million new cases reported in 2020 and an estimated 769,000 deaths, accounting for 1 in every 13 cancer-related deaths worldwide. GC ranks fifth in incidence and fourth in mortality globally [1,2]. The mortality rate among GC patients is closely linked to the stage of the disease at diagnosis. GC can be classified into 2 main topographic subsites: cardia GC, which originates in the area of the stomach adjacent to the esophagogastric junction, and non-cardia GC, which arises from more distal regions of the stomach. These subsites differ in their descriptive epidemiology and risk factor profiles [3]. Among all clinical treatments for GC, surgery remains the only approach capable of completely eradicating the disease and serves as the cornerstone of GC management [4,5]. Accurate preoperative staging and resectability assessment are critical for successful surgical outcomes. However, traditional diagnostic methods such as gastrointestinal imaging, computed tomography with abdominal enhancement, and general gastroscopy often fall short in precisely identifying the nature and location of lesions or detecting the presence of distant metastases [6]. The success of a gastrectomy procedure hinges on the precise intraoperative differentiation between malignant lesions and adjacent gastric tissue, enabling both complete tumor excision and accurate delineation of surgical margins. This highlights the need for advanced imaging techniques to address these challenges effectively.

Precision surgery aims to achieve a balance between maximizing the resection of targeted tissues and preserving as much normal tissue as possible [7]. The advent of advanced techniques such as endoscopic and fluorescence imaging has significantly propelled the development of precision surgery [8,9]. Among these, fluorescence imaging-guided surgery aligns seamlessly with the principles of precision surgery. By visualizing targeted tissues, surgeons can accurately delineate tumor boundaries or diseased areas to ensure complete resection, while simultaneously visualizing normal tissues to preserve organ function. Among these, fluorescence imaging-guided surgery aligns seamlessly with the principles of precision surgery. By visualizing targeted tissues, surgeons can accurately delineate tumor boundaries or diseased areas to ensure complete resection, while simultaneously visualizing normal tissues to preserve organ function [10]. In recent years, near-infrared (NIR) fluorescence imaging has emerged as a vital tool in image-guided tumor surgeries due to its inherent advantages, including excellent tissue penetration, minimal tissue absorption and light scattering, and low autofluorescence [11]. Among the available agents, indocyanine green (ICG) has received Food and Drug Administration (FDA) approval for various clinical applications, while fluorescein and methylene blue (MB) have also demonstrated their efficacy in achieving high-precision surgery. The FDA approved pafolacianine (Cytalux) in November 2021 for use in ovarian cancer surgeries and later expanded for lung cancer surgeries [12,13]. Furthermore, fluorescein and MB have been efficiently used for high-precision surgery [14]. The fluorescence imaging technique holds great promise as a routine intraoperative method during GC surgery, as it enables clear visualization of surgical margins and the detection of small occult lesions. However, there is currently a lack of NIR fluorescent contrast agents specifically designed to target stomach and gastric tumors. Consequently, the development of NIR fluorescent contrast agents with high diagnostic and therapeutic potential is critical for advancing intraoperative gastroendoscopy and improving outcomes in gastric tumor surgeries.

Our previous study demonstrated that MB can directly target the stomach in mice [15]. Specifically, MB was shown to increase gastric acid secretion by activating the H^+^/K^+^ ATPase, which is likely associated with this proton pump [16]. Building on this, we synthesized ESS65-Cl, a phenoxazine derivative similar to MB. Based on its physicochemical properties, we hypothesize that ESS65-Cl may also target gastric tissue through mechanisms related to H^+^/K^+^ ATPase activity. In parallel, IR-780, a lipophilic cationic heptamethine dye, has demonstrated cancer imaging capabilities through selective accumulation in mitochondria via an anion transporting polypeptide transporter [17]. Additionally, the selective tumor uptake of IR-780 has been shown to depend on cellular energy metabolism and plasma membrane potential, but is independent of endocytosis, mitochondrial membrane potential, and ATP-binding cassette transporters [18]. IR-780 can also passively accumulate in tumors via the enhanced permeability and retention effect [19,20]. Nonetheless, the precise mechanisms underlying its tumor-targeting properties remain incompletely understood.

In this study, we investigated the potential of 2 NIR dyes, ESS65-Cl and IR-780 [17,21], as effective imaging and targeting agents for intraoperative imaging of gastric tissues and tumors. These dyes demonstrated dual-channel imaging capabilities in gastric animal models, offering distinct visualization of gastric tissue and tumor boundaries. This dual-channel imaging technique addresses the clinical challenges of determining the proximal esophageal margin and defining the gastrectomy scope in gastric tumor surgeries. By leveraging complementary positive and negative imaging effects, this approach enhances precision and accuracy during surgical procedures.

## Materials and Methods

IR-780 was purchased from KE Biochem (Shanghai, China). ESS65-Cl was synthesized according to the previously reported methods with slight modifications [22,23]. All chemicals and solvents used were of American Chemical Society grade. The purity was determined using Waters liquid chromatography–mass spectrometry (LC-MS), which included an Alliance e2695 separation module, a 2998 PDA detector (212 to 800 nm), and an Acquity QDA detector (*m*/*z* range: 50 to 1,239). An XBridge C18 (4.6 × 150 mm, 5 μm) reverse-phase high-performance liquid chromatography column (Waters) was used for LC-MS analysis.

### Measurement of optical properties

The NIR fluorescence imaging system [16,24] was utilized to evaluate the optical properties and fluorescence intensity of the NIR fluorophores. Absorbance and fluorescence measurements were conducted using fiber optic HR2000 spectrometers (200 to 1,100 nm) from Ocean Optics (Dunedin, FL). The pH stability of ESS65-Cl was examined in various buffer solutions, and its photostability was assessed at multiple concentrations. These evaluations are crucial for ensuring the reliability and suitability of ESS65-Cl in fluorescence imaging applications.

### Cell culture and cell viability

Human gastric epithelial cell lines (GES-1; RRID: CVCL_EQ22) and human GC cell lines (SGC-7901; RRID: CVCL_0520) were obtained from the Cell Bank of the Committee on Type Culture Collection of the Chinese Academy of Sciences (Shanghai, China).

Short tandem repeat profiling revealed that the SGC-7901 cells used in this study are genetically homologous to HeLa cells [25,26]. GES-1 cells were cultured in Dulbecco's modified eagle medium supplemented with 10% fetal bovine serum and ampicillin/streptomycin, while SGC-7901 cells were maintained in RPMI 1640 medium under a 5% CO₂ humidified atmosphere at 37 °C. Both cell lines were incubated with varying concentrations (0.01, 0.1, 1, 10, and 100 μM) of ESS65-Cl or IR-780 for 60 min. Cell viability was assessed using the MTT (3-[4,5-dimethylthiazol-2-yl]-2,5 diphenyl tetrazolium bromide) assay to evaluate the potential cytotoxicity of the fluorophores.

### Fluorescence imaging

GES-1 and SGC-7901 cells were incubated with ESS65-Cl (10 μM) or IR-780 (10 μM) for 24 h at 37 °C. NIR fluorescence imaging was performed using the K-FLARE system, a dual-channel platform that provides white-light color images (400 to 650 nm) alongside 2 independent fluorescence channels at 700 and 800 nm. Excitation at 630 nm (fluence rate: 2 mW/cm^2^) was applied for ESS65-Cl in the 700-nm channel, while excitation at 760 nm (fluence rate: 4 mW/cm^2^) was used for IR-780 in the 800-nm channel. For merged NIR images, fluorescence signals from the 700- and 800-nm channels were pseudo-colored red and green, respectively. Fluorescence imaging of the cells was performed using a confocal microscope (Leica SP8, Germany). The fluorescence intensity of GES-1 and SGC-7901 cells treated with ESS65-Cl or IR-780 was analyzed using ImageJ v1.8.0 software (National Institutes of Health, Bethesda, MD).

### In vivo biodistribution and clearance of NIR fluorophores

The work has been reported in accordance with the ARRIVE guidelines (Animal Research: Reporting of In Vivo Experiments) [27]. All animal experiments were conducted under the Institutional Animal Care and Use Committee at Xi’an Jiaotong University (No. 2021-094) and Mass General Hospital (No. 2016N000136). All mice were housed in cages with freely available food and water supplies for 1 week, and light/dark cycles (12 h/12 h) and temperature (23 to 25 °C) were maintained to standardize the animal experimental conditions. Two hundred nanomoles of ESS65-Cl (100 μl) or 100 nmol of IR-780 (100 μl) in saline containing 10% bovine serum albumin (BSA) was administered intravenously to the mice. NIR fluorescence signals were observed 4 h post-injection using the NIR fluorescence imaging system to evaluate the biodistribution and clearance of ESS65-Cl and IR-780 in vivo. This short-term biodistribution provides initial tissue uptake and clearance, and proof of specificity. Following imaging, the mice were euthanized, and their major organs were dissected for fluorescence imaging. Three mice were analyzed for each sample group.

### Animal models and intraoperative dual-channel imaging

To establish a subcutaneous gastric tumor model, 1 × 10^7^ SGC-7901 cells suspended in 200 μl of saline/Matrigel were inoculated subcutaneously into the left flank of the male nude mice (8-week-old male; 20 to 25 g). For the orthotopic gastric tumor model, the mice were anesthetized, and the abdomen was opened. Then, 1 × 10^7^ SGC-7901 cells suspended in 100 μl of saline/Matrigel were injected directly between the submucosa and muscular layer of the stomach, and the wound was sutured following the previously described method [28]. When the orthotopic gastric tumor diameter reached 0.3 cm or abdominal nodules were palpable, 200 nmol of ESS65-Cl (100 μl) was injected intravenously into the gastric tumor-bearing mice, and NIR fluorescence signals were observed at 1, 2, 4, 24, and 48 h post-injection. IR-780 (100 nmol in 100 μl) was administered 72 h prior to the initiation of NIR fluorescence imaging. After completing the simultaneous intraoperative dual-channel imaging, the mice were euthanized, and major organs and gastric tumor tissues were dissected for fluorescence imaging. Three mice were analyzed for each sample.

### Quantitative analysis of fluorescence images

ImageJ v1.8.0 was used to quantify the fluorescence and background intensities of the region of interest (ROI) in each tissue. The following steps demonstrate the calculation of the signal-to-background ratio (SBR) [29]:

$$\text{SBR}=\frac{I_{\text{ROI}}}{I_{\text{Auto}}}$$ (1)

where the average intensity of an ROI is represented by *I*_ROI_ and the intensity of the muscle was denoted by *I*_Auto_. The same formula was applied to calculate the tumor-to-background ratio (TBR) and *I*_T_ represented the intensity of the tumor tissue.

$$\text{TBR}=\frac{I_{T}}{I_{\text{Auto}}}$$ (2)

### Fluorescence imaging and hematoxylin and eosin staining

Excised organs, stomach, and gastric tumor tissues were trimmed and embedded in Optimal Cutting Temperature compound. Frozen sections (10 μm) were then cut using a cryostat (Leica, Germany). Fluorescence imaging was performed on the sections using a ZEISS ZEN2 microscope, while one part of the serial sections was stained with hematoxylin and eosin (H&E). Finally, images of the sectioned slides were captured using a Leica microscope (20× objective).

### Statistical analysis

A 2-tailed paired or unpaired Student’s *t* test was performed on Prism 8 software (GraphPad, San Diego, CA). Results were presented as the mean ± SEM for all the image analyses on the NIR imaging system and confocal microscope. A value of *P <* 0.05 was considered to be statistically significant.

## Results

### Optophysical properties of NIR fluorophores ESS65-Cl and IR-780

**Fig. 1.**
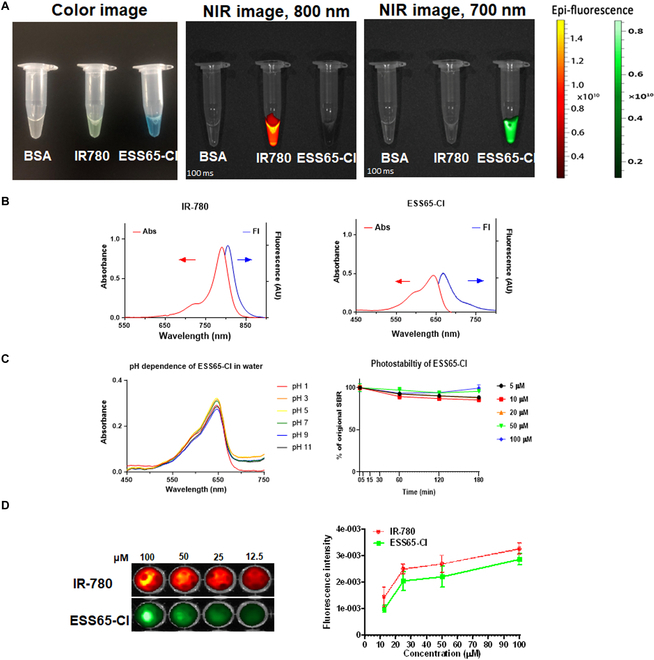
Optophysical properties of NIR fluorophores. (A) Optical properties of ESS65-Cl and IR-780 in 10% BSA (100 μM) under the NIR fluorescence imaging system. Excitation at 630 nm was applied for ESS65-Cl in the 700-nm channel, while excitation at 760 nm was used for IR-780 in the 800-nm channel. (B) Representative absorption and fluorescence spectra of ESS65-Cl and IR-780 in 10% BSA (5 μM). (C) pH stability of ESS65-Cl in various buffer solutions and photostability of ESS65-Cl in various concentrations. (D) Fluorescence intensity of ESS65-Cl and IR-780 in 10% BSA (12.5, 25, 50, and 100 μM) was also analyzed by the NIR fluorescence imaging system.

Upon measuring the optical and physicochemical properties of these NIR fluorophores (Fig. S1), ESS65-Cl exhibited absorbance and fluorescence spectra in the 700-nm channel, which is approximately 140 nm lower than the corresponding 800-nm emitting fluorophore, IR-780. This allows for simultaneous dual-channel imaging without spectral overlap (Fig. 1A and B). Next, the stability of ESS65-Cl was assessed in various conditions. The fluorophore ESS65-Cl demonstrated stability across a wide pH range (Fig. 1C), including in strongly acidic conditions (e.g., pH 1.0) and slightly basic conditions (i.e., pH 9.0). We also assessed the photostability of ESS65-Cl by irradiating it with 660-nm NIR light at a power density of 1 mW cm^−2^ for 3 h using the K-FLARE imaging system (Fig. 1C). ESS65-Cl demonstrated exceptional photostability at concentrations ≥20×10^−6^ M. Additionally, the fluorescence intensity of ESS65-Cl and IR-780 was enhanced by increasing the fluorophore concentrations (12.5, 25, 50, and 100 μM) (Fig. 1D).

### Fluorescence imaging of NIR fluorophores IR-780 and ESS65-Cl

**Fig. 2.**
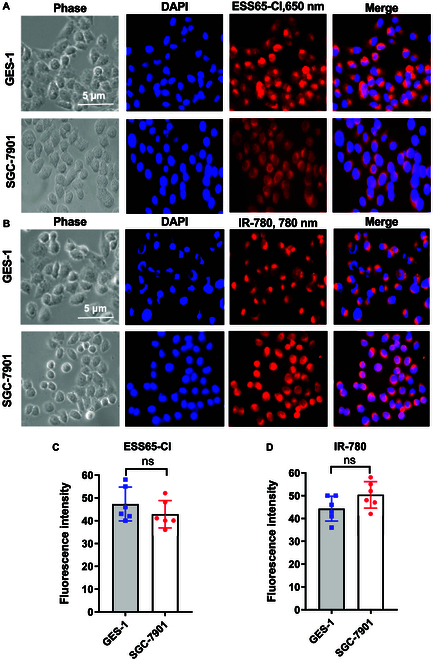
Fluorescence imaging of NIR fluorophores (ESS65-Cl and IR-780) in human gastric epithelial GES-1 cells and gastric cancer SGC-7901 cells. (A) GES-1 and SGC-7901 cells were incubated with ESS65-Cl (10 μM) for 24 h at 37 °C, and GES-1 and SGC-7901 cells were observed under the confocal microscope. (B) GES-1 and SGC-7901 cells were incubated with IR-780 (10 μM) for 24 h at 37 °C, and GES-1 and SGC-7901 cells were observed under the confocal microscope. (C) Fluorescence intensity of GES-1 and SGC-7901 cells treated with ESS65-Cl was analyzed using ImageJ. (D) Fluorescence intensity of GES-1 and SGC-7901 cells treated with IR-780 was analyzed using ImageJ. Error bars show means ± SEM.

The specificity of ESS65-Cl and IR-780 for the epitope of living cells was tested using human gastric epithelial cells (GES-1) and human GC cells (SGC-7901). As shown in Fig. 2A, ESS65-Cl produced strong fluorescence in GES-1 cells and weaker signals in SGC-7901 cells (Fig. 2A), whereas IR-780 displayed the opposite trend (Fig. 2B). Thus, in vitro, ESS65-Cl preferentially labeled gastric epithelial cells, while IR-780 targeted GC cells. The fluorescence intensity of GES-1 and SGC-7901 cells treated with ESS65-Cl or IR-780 was analyzed using ImageJ, and no significant difference was observed (*P* > 0.05) (Fig. 2C and D). Moreover, cell viability treated with ESS65-Cl and IR-780 decreased with the increase of fluorophore concentrations (Fig. S2).

### Biodistribution patterns of NIR fluorophores ESS65-Cl and IR-780

To evaluate in vivo biodistribution and clearance, ESS65-Cl and IR-780 were injected intravenously into CD-1 mice and imaged under the K-FLARE imaging system at 0.5 to 48 h post-injection (Fig. S3). ESS65-Cl showed a relatively rapid distribution into the stomach and fast renal excretion at 4 h post-injection, which together lower nonspecific background tissue uptake and retention [22]. In comparison, IR-780 showed higher uptake in the major organs including stomach, heart, liver, lungs, and other organs, due to its hydrophobic character, protein binding, and lung microvasculature trapping. Its relatively longer distribution and elimination of half-life values, and slow hepatobiliary excretions reflect more chance of exposure to the bloodstream and tumoral tissue [30].

### Simultaneous intraoperative dual-channel imaging of subcutaneous gastric tumor in vivo

**Fig. 3.**
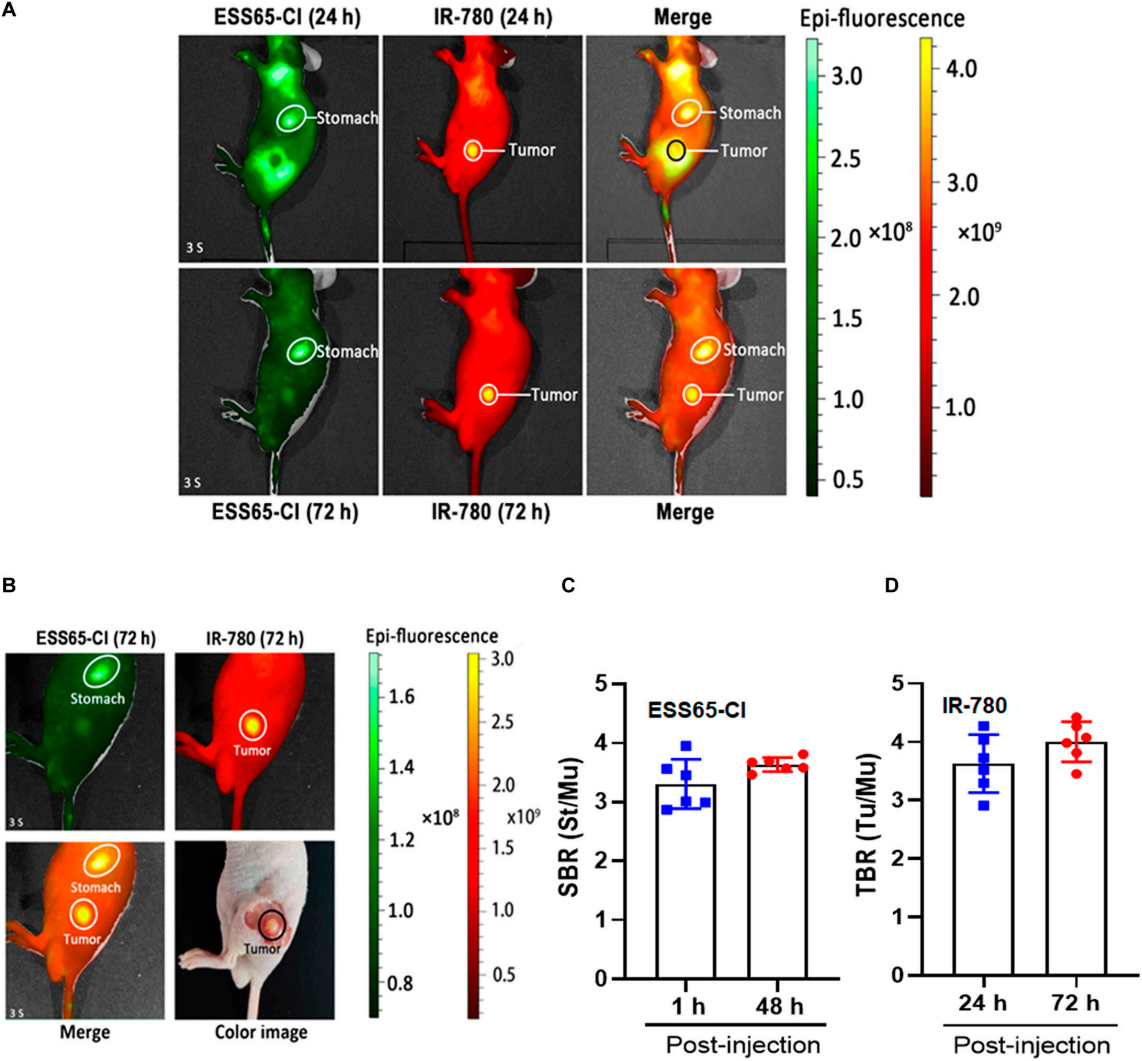
Targeted imaging of IR-780 in subcutaneous gastric tumor of nude mice at 24 h and 72 h post-injection, and targeted imaging of ESS65-Cl in the stomach of nude mice at the same time point. One hundred nanomoles of IR-780 (100 μl) was injected intravenously and observed up to 24 h post-injection, and then 200 nmol of ESS65-Cl (100 μl) was injected intravenously 1 h prior to imaging. (A and B) Simultaneous intraoperative dual-channel imaging of gastric tumor (800 nm channel) and stomach (700 nm channel). (C) The signal-to-background ratio (SBR) was calculated by comparing the signals of the stomach against the surrounding muscle. (D) TBR was calculated by comparing the signals of the tumor against the surrounding muscle. Six mice were analyzed for each sample. Error bars show means ± SEM.

After injecting NIR fluorophores (ESS65-Cl and IR-780) into the established mouse model of subcutaneous gastric tumors, real-time NIR targeted imaging was performed up to 72 h post-injection. Subcutaneous gastric tumors were successfully detected using IR-780 at the 800-nm channel, while stomach imaging was captured at 700 nm following ESS65-Cl injection. As shown in Fig. 3A, ESS65-Cl was injected 24 h after IR-780, and fluorescent signals in both the tumors and stomach were simultaneously monitored. Significant NIR fluorescence signals were detected in the gastric tumor, with the strongest fluorescence observed 48 h post-injection of IR-780. Meanwhile, in vivo biodistribution and clearance of ESS65-Cl suggested that ESS65-Cl can target the stomach in CD-1 mice (Fig. S3). In contrast, strong NIR fluorescence signals were noted in the stomach, with the highest fluorescence observed 24 h after the injection of ESS65-Cl (Fig. S4). Simultaneous intraoperative dual-channel imaging of the gastric tumor (800 nm IR-780) and stomach (700 nm ESS65-Cl) is shown in Fig. 3B. The SBR of the stomach 48 h post-injection of ESS65-Cl was slightly higher than at 1 h (Fig. 3C). The TBR of the gastric tumor 72 h post-injection of IR-780 was marginally higher than at 24 h (Fig. 3D).

### Dual-channel imaging in subcutaneous gastric tumor with ESS65-Cl and IR-780

**Fig. 4.**
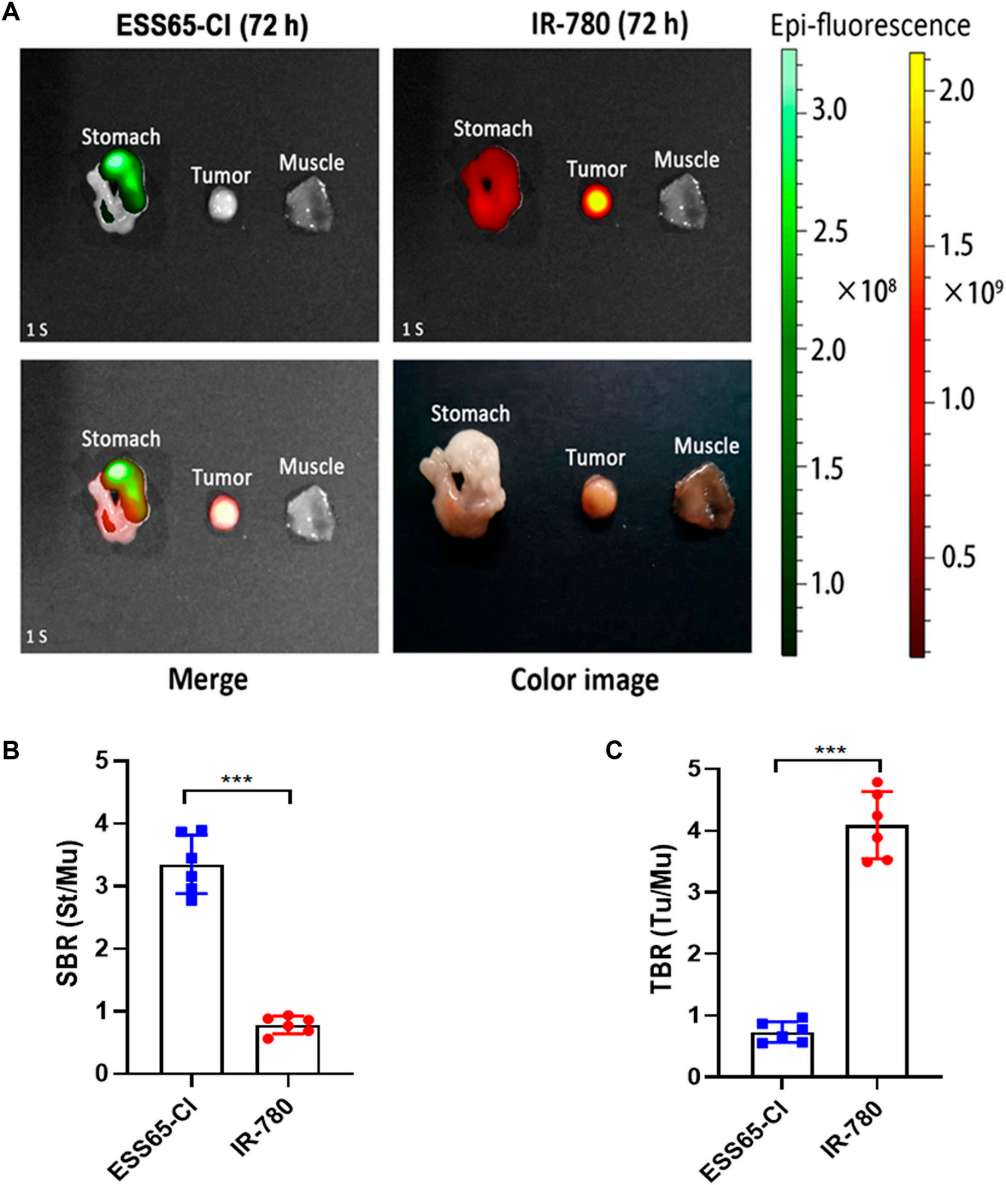
Dual-channel imaging in subcutaneous gastric tumor with ESS65-Cl and IR-780. (A) NIR imaging of the resected stomach and tumor with post-injection of ESS65-Cl (700 nm channel) and IR-780 (800 nm channel). (B) SBR of the resected stomach and (C) TBR of the resected tumor. Six mice were analyzed for each sample. Error bars show means ± SEM. ***P < 0.001.

Dual-channel imaging of resected tissues and organs was performed using ESS65-Cl and IR-780. As shown in Fig. 4A, fluorescence signals from the resected stomach at 48 h post-injection and from the resected tumor at 72 h post-injection demonstrated significantly higher fluorescence intensity compared to muscle tissue. The SBR in the stomach was consistently and significantly enhanced following ESS65-Cl injection and reached 3.3 at 48 h post-injection (Fig. 4B). Additionally, the TBR in the tumor was notably increased after IR-780 injection (TBR = 4.0, Fig. 4C). Interestingly, strong NIR fluorescence signals were also observed in the lung (Fig. S5). Polymethine NIR fluorophores such as IR780 may show lung accumulation due to several physicochemical and biological factors, including high hydrophobicity and lipophilicity, aggregation in circulation, protein binding and lung microvasculature trapping, and passive entrapment in pulmonary capillaries [17,21].

### Simultaneous intraoperative dual-channel imaging of orthotopic gastric tumor in vivo

**Fig. 5.**
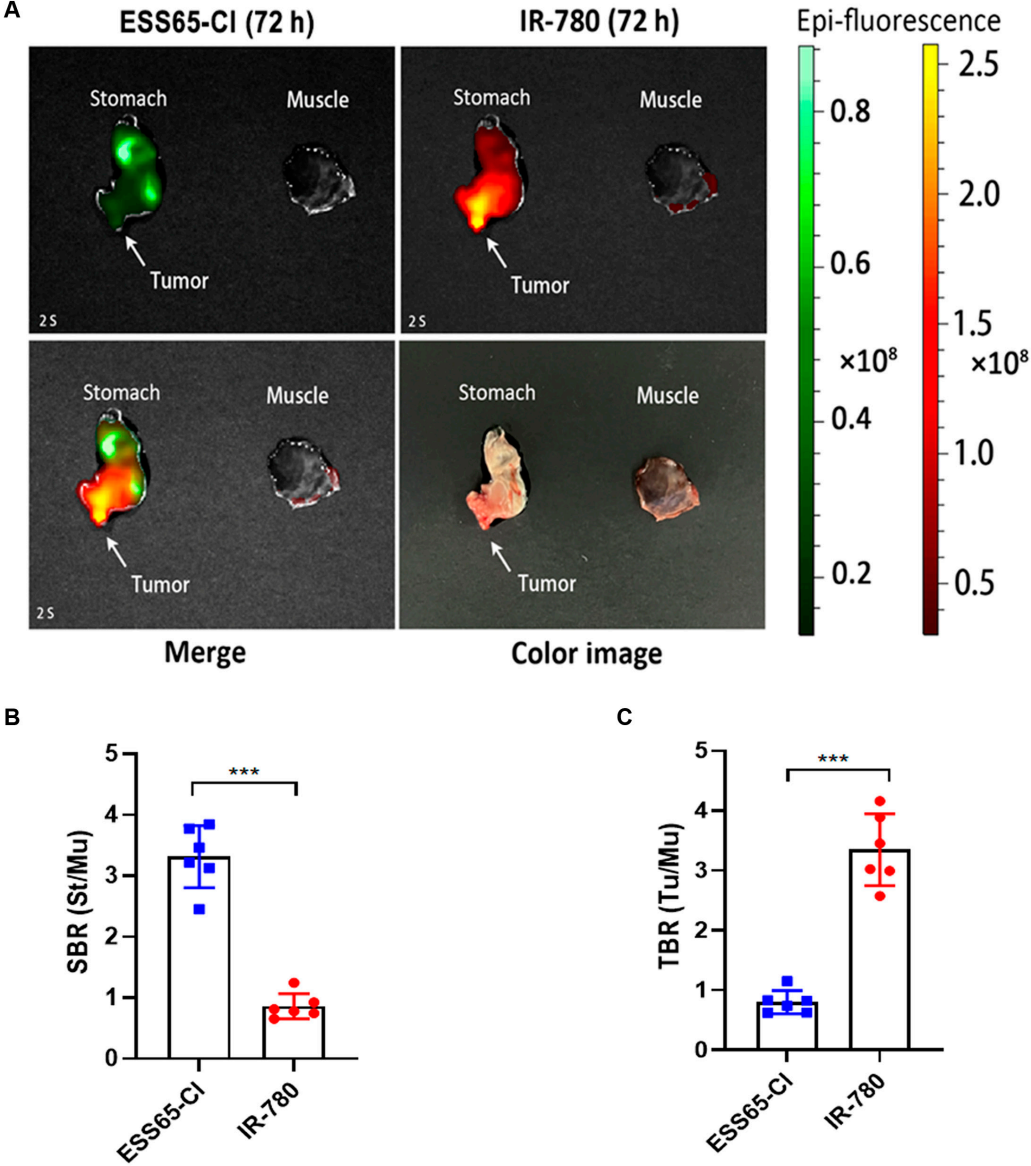
Dual-channel imaging in orthotopic gastric tumor with ESS65-Cl and IR-780. (A) NIR imaging of the resected tumor and stomach with post-injection of ESS65-Cl (700 nm channel) and IR-780 (800 nm channel). (B) SBR of the resected stomach and (C) TBR of the resected tumor. Six mice were analyzed for each sample. Error bars show means ± SEM. ***P < 0.001.

Dual-channel imaging was explored in orthotopic gastric tumor-bearing nude mice. Orthotopic gastric tumors were successfully visualized using IR-780 at 800 nm, while stomach imaging was captured at 700 nm after the injection of ESS65-Cl (Fig. 5A). Fluorescence signals from the resected stomach at 48 h post-injection and from the resected tumor at 72 h post-injection exhibited significantly higher intensity compared to muscle tissue. The SBR of ESS65-Cl and TBR of IR-780 was consistently enhanced, both reaching 3.3. As shown in Fig. S6, ESS65-Cl demonstrated strong targetability to the stomach, while IR-780 exhibited strong targetability to the orthotopic gastric tumor, with some NIR fluorescence signals observed in the lung (Fig. S7).

### Fluorescence imaging of NIR fluorophores and histological analysis

**Fig. 6.**
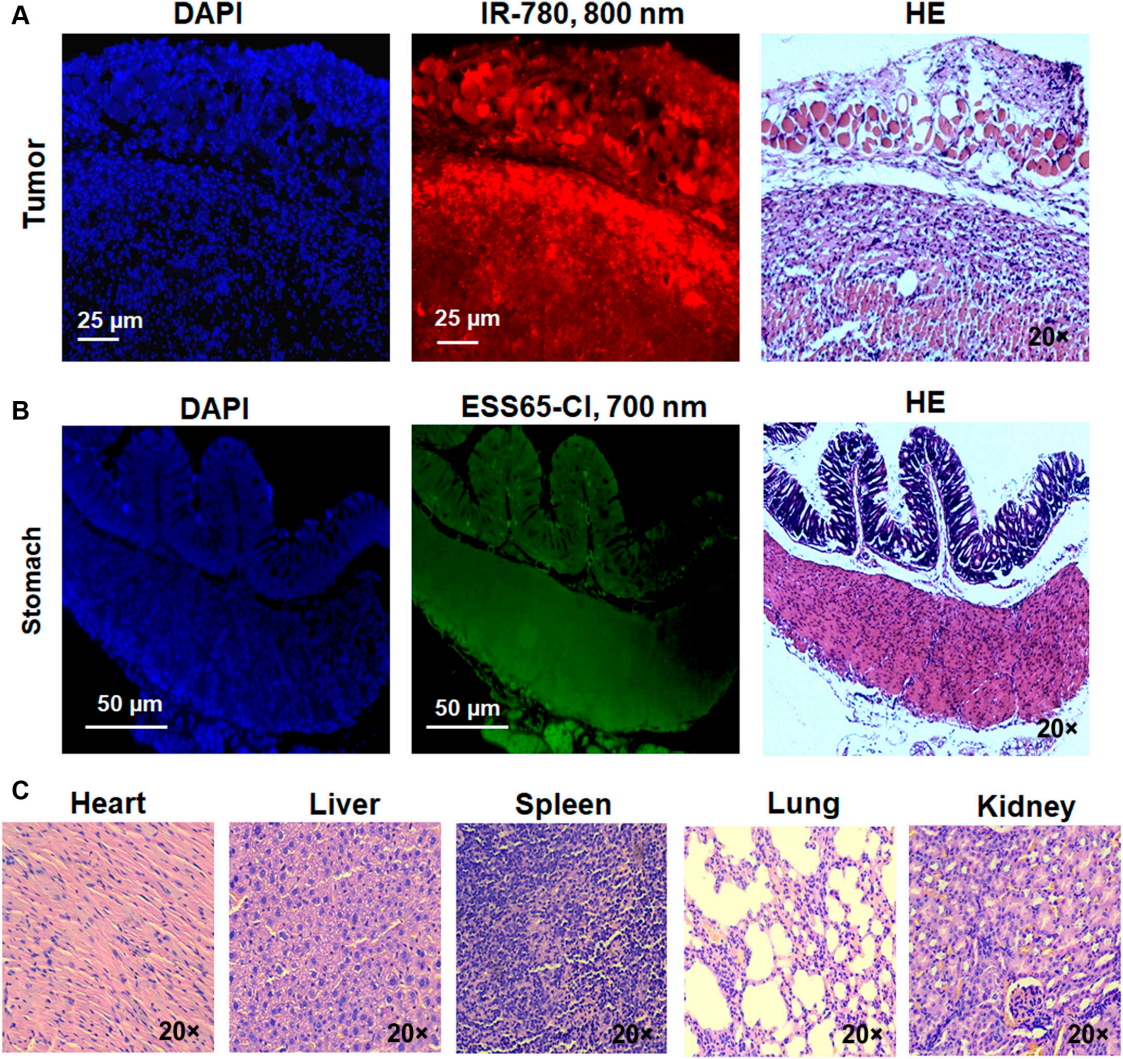
Microscopic images of tumor, stomach, and major organs.ESS65-Cl and IR780 were injected intravenously into nude mice bearing gastric tumor 72 h prior to resection. NIR fluorescence and H&E images of tumor (A) and stomach (B) were displayed along with DAPI staining. (C) The resected major organs (heart, liver, spleen, lung, and kidney) were also H&E stained and imaged under a ZEISS ZEN2 microscope and a Leica microscope.

To further assess the role of ESS65-Cl and IR-780 in dual-channel imaging, histological detection and analysis were performed. Pathological examination of the gastric tumor at 72 h post-injection of IR-780 revealed red fluorescence signals in the gastric tumor tissues (Fig. 6A). Additionally, a pathological examination of gastric tissues at 48 h post-injection of ESS65-Cl displayed green fluorescence signals, with no observed pathological damage to the gastric tissues (Fig. 6B). No apparent tissue or cellular damage was detected in the major organs (heart, liver, spleen, lung, and kidney) (Fig. 6C). This study demonstrated that ESS65-Cl exhibited specificity for gastric tissues, while IR-780 showed strong targeting ability for gastric tumor tissues. Therefore, dual-channel imaging proves valuable for targeting both gastric tissues and tumors.

## Discussion

Surgical resection remains the primary treatment for GC, requiring high precision to maximize oncological efficacy while minimizing functional impairment [31]. Fluorescence-guided surgery enhances this precision, with ICG serving as a clinically approved NIR contrast agent [32]. However, ICG has several inherent drawbacks, including low stability in aqueous environments, rapid blood clearance, low quantum yield, and poor organ specificity [33]. To address these limitations, we developed ESS65-Cl, a gastric tissue-targeting agent, and IR-780, a tumor-targeting agent [34,35], as paired imaging agents for the simultaneous detection of gastric tissues and tumors. This approach enhances the precision of tumor resection while minimizing the risk of iatrogenic injury.

ESS65-Cl emits at 700 nm, offering low autofluorescence, minimal tissue interference, and high specificity for gastric tissues. In contrast, IR-780 (800 nm) exhibits strong tumor accumulation, excellent stability, and high fluorescence intensity. In vitro, ESS65-Cl selectively labeled gastric epithelial cells (GES-1), while IR-780 targeted GC cells (SGC-7901), confirming their complementary roles. In the present study, the viability of GES-1 and SGC-7901 cells decreased with increasing concentrations of ESS65-Cl and IR-780. Notably, treatment with 10 μM of either probe reduced cell viability to approximately 50%. Previous studies have reported that exposure to 10 μM IR-780 lowers cell viability to below 80% in breast cancer cells [35,36]. Variations in the cytotoxicity of the same fluorescent probe across different cell lines are expected and may be attributed to the intrinsic biological characteristics of each cell type, including metabolic capacity [37], membrane transport proteins [38], antioxidant ability [39], cell damage repair ability [40], and cell proliferation [41]. Therefore, to minimize cytotoxicity, future cellular and imaging experiments should employ lower concentrations of ESS65-Cl and IR-780.

In vitro studies are conducted under simplified conditions, without the complex physiological environments and dynamic processes present in vivo. As a result, 2 probes may exhibit similar in vitro characteristics yet show distinct targeting behaviors in vivo. Such differences arise from variations in physicochemical properties (e.g., hydrophilicity/lipophilicity, charge, polar surface area, and stability) and physiological factors encountered in vivo (e.g., metabolism, biological barriers, and tissue microenvironment) [19,20]. This dual-channel approach enables surgeons to delineate tumor margins (IR-780) and surrounding gastric structures (ESS65-Cl) in real time, improving resection accuracy and reducing iatrogenic injury.

In preclinical gastric tumor models, ESS65-Cl precisely marked gastric tissue boundaries, while IR-780 outlined tumor margins. This is particularly relevant for gastroesophageal junction adenocarcinomas, where surgical standardization remains challenging due to anatomical and biological variability. The affinity of ESS65-Cl for the murine forestomach (a functional analog to human gastric storage regions) further supports its translational potential, though interspecies differences must be considered.

Dual-channel imaging allows the surgical area to be observed simultaneously from 2 different perspectives or using 2 distinct fluorescent characteristics. This multidimensional imaging method enables surgeons to gain a more comprehensive understanding of the tumor’s location, size, shape, and its relationship with surrounding tissues. It aids in making more accurate judgments and formulating refined surgical plans during the procedure [42]. However, there remains no consensus on its origin or distinctive biological features [43], which complicates the establishment of standardized therapeutic strategies. These strategies include perioperative treatment, surgical approach, lymph node dissection, and the extent of esophageal resection [44]. The mouse stomach consists of 2 regions: the glandular stomach and the nonglandular or fore-stomach [45]. Our study demonstrates that while ESS65-Cl does not specifically target gastric tumors, it exhibits a strong preference for the fore-stomach, which serves as a temporary site for food storage and digestion. Dual-channel imaging holds great promise for enhancing precision in gastrointestinal surgery via 2 distinct fluorescent characteristics of ESS65-Cl and IR-780. First, using the signal ratio of the 2 channels normalizes probe distribution bias, which sharpens the true tumor boundaries. This approach addresses the challenge of heterogeneous cell density and improves the accuracy of intraoperative margin assessment. Second, the complementary characteristics of the probes reduce false-positive signals and enhance boundary clarity in both subcutaneous and orthotopic gastric tumor models. Finally, in future work, we plan to quantify boundary deviation distances by comparing single-probe and dual-channel imaging results with pathological sections, and to perform 3D reconstructions from serial section imaging to assess volumetric deviations between the 2 approaches.

Several translational challenges remain. While murine models are valuable, their physiological differences from humans, such as gastric anatomy (glandular vs. forestomach) and immune responses, limit direct extrapolation [45]. Larger animal models (e.g., pigs and dogs) may better approximate human gastric surgery [46,47]. Additionally, clinical adoption of dual-channel imaging requires addressing several barriers: (a) Dual-channel systems should integrate seamlessly with existing surgical tools, such as laparoscopic cameras or robotic platforms, through modular add-ons to avoid disrupting the surgical workflow. (b) Exogenous contrast agents (e.g., ESS65-Cl and IR-780) must undergo extensive safety profiling. (c) Accurate co-registration across tissue types and surgical conditions (e.g., bleeding or motion) is vital. Miniaturization for minimally invasive procedures, such as laparoscopic or robotic surgeries, presents additional engineering challenges. Meanwhile, artificial intelligence (AI)-driven data synthesis (e.g., highlighting tumor margins) can reduce the risk of information overload. (d) Early involvement of surgeons in the system design phase will foster trust and encourage adoption. Additionally, ethical concerns related to liability in interpreting imaging data will require the development of clear guidelines and targeted training programs. By addressing these integration strategies and translational challenges through iterative design, stakeholder collaboration, and robust clinical validation, dual-channel imaging has the potential to significantly enhance surgical precision while aligning with real-world clinical needs.

For future directions, dual-channel imaging could revolutionize early GC diagnosis, where white-light endoscopy misses 10% to 30% of cases [5,48]. Dual-channel imaging could fill this diagnostic gap by integrating multidimensional data and AI, thereby improving diagnostic sensitivity and specificity. Potential advancements include the following: (a) miniaturized endoscopes with NIR capabilities for hard-to-reach regions, (b) AI-assisted analysis to differentiate malignant vs. benign lesions in real time, and (c) enhanced endoscopic submucosal dissection by improving margin assessment. By overcoming these challenges, dual-channel imaging aligns with global “early detection and treatment” strategies, offering a transformative tool for precision oncology.

In summary, ESS65-Cl and IR-780 can be specifically taken up by gastric epithelial cells and cancer cells, respectively. ESS65-Cl is expected to be a beneficial NIR agent for addressing the long-standing controversy surrounding the proximal esophageal resection margin and the extent of gastric resection in proximal GC. When used together, ESS65-Cl and IR-780 enable dual-channel NIR fluorescence imaging, allowing for the simultaneous and accurate intraoperative identification of gastric tissues and tumors, with distinct colors, in both subcutaneous and orthotopic gastric tumor xenograft models. Therefore, it holds significant potential for guiding the identification and navigation of the proximal esophageal margin and the gastrectomy area during GC surgery.

## Data Availability

The datasets generated and/or analyzed during the current study are available from the corresponding authors on reasonable request.
