# Supplementary material for: Dual-Channel NIR Fluorescence Imaging for Precise Delineation of Gastric Tumor Margins
Source: Biomater Res. 2025 Oct 28;29:0275. doi: 10.34133/bmr.0275 (PMC12559798; doi:10.34133/bmr.0275)
Supplement: Supplementary 1 — Supplementary Methods Figs. S1 to S7 [file bmr.0275.f1.docx]

**Supporting Information**

**Supplementary Methods:** A mixture of 3-((2-chloroethyl)(methyl)amino)phenol (Compound 1, 100mg, 0.54 mmol), 3-methoxy-*N,N*-dimethyl-4-nitrosoaniline (Compound 2, 97mg, 0.54 mmol) and HClO_4_ acid (0.54 mmol) in 90% i-PrOH/H_2_O (10 mL) was stirred at reflux for 4h in the dark (the reaction progress was monitored by HPLC). The dark-blue solution was evaporated, and the residue was purified by using Waters preparative HPLC consisting of a 2489 UV/Visible detector, a 1525 Binary HPLC pump, and an XBridge Prep C18 (19×150 mm, 5 µm) reverse-phase HPLC column (MeOH/H_2_O, 5-95%). The eluent was collected, concentrated by rotary evaporation, and dried under vacuum overnight to give the blue solid, ESS65-Cl, 71mg, 42% yield. MS (ESI) calculated for the chemical formula C_17_H_19_ClN_3_O^+^, exact mass 316.12, found 316.19 [M]^+^. 1H-NMR (500 MHz, D_2_O): δ ppm 7.75 (m, 2H, Ar-H), 7.39, (m, 2H, Ar-H), 6.92, (m, 2H, Ar-H), 4.05 (m, 2H), 3.84 (m, 2H), 3.21 (S, 6H), 3.12 (S, 3H). 13C-NMR (125 MHz, CD_3_OD): δ ppm 159.98, 159.06, 150.88, 150.46, 136.99, 135.60, 135.11, 135.00, 119.60, 118.16, 97.93, 97.66, 55.31, 42.06, 41.97, 40.62, 9.23.


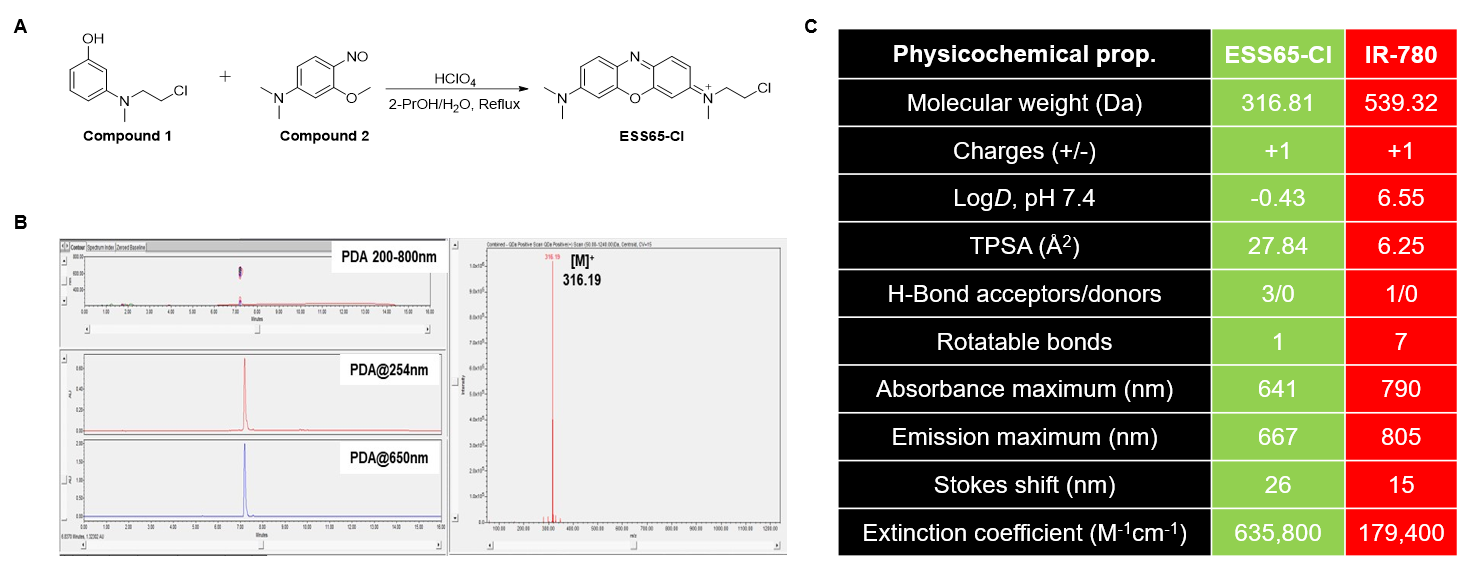


**Figure S1.** The synthetic process and physicochemical properties of ESS65-Cl and IR-780. (A) The synthetic route of ESS65-Cl. (B) The HPLC spectrum of ESS65-Cl. (C) Physicochemical properties of ESS65-Cl and IR-780.


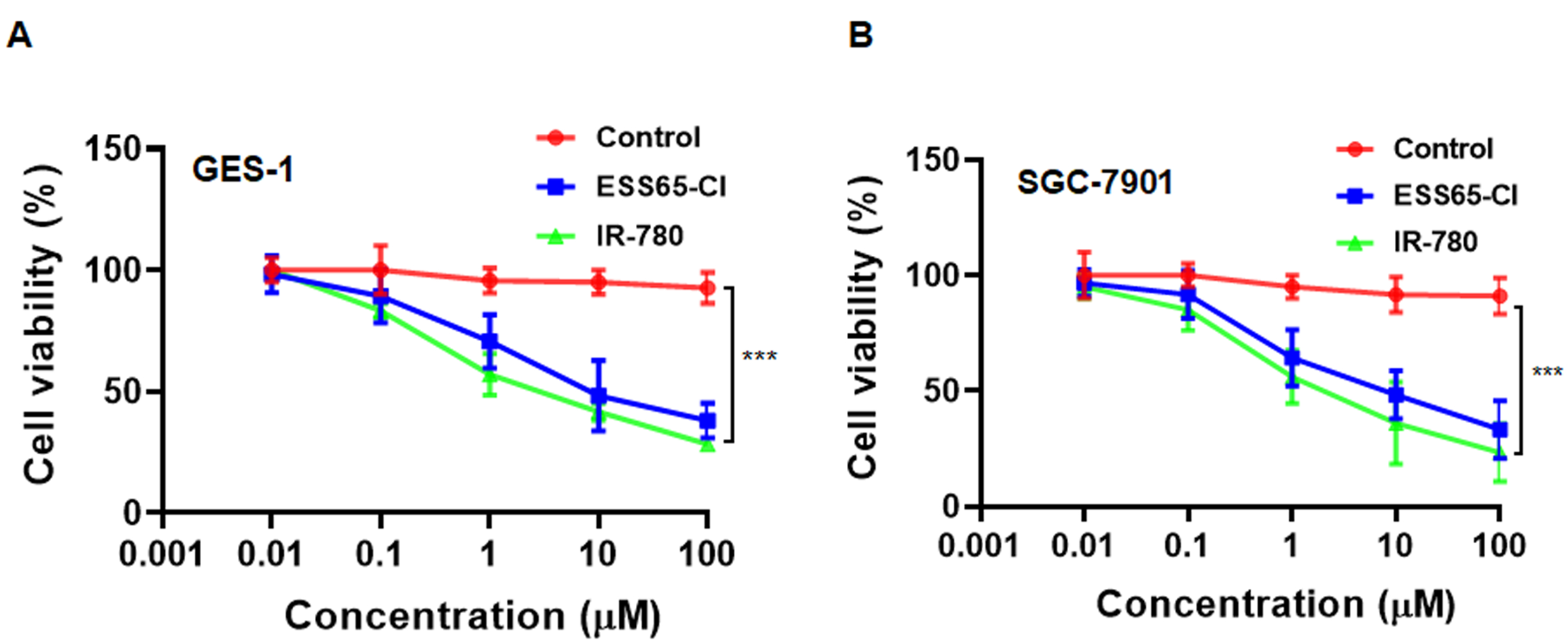


**Figure S2.** Cell viability treated with ESS65-Cl and IR-780. Human gastric epithelial GES-1 cells and gastric cancer SGC-7901 cells were incubated with ESS65-Cl and IR-780 with different concentrations for 60 minutes, and cell viability was determined compared to a control sample (GES-1 and SGC-7901 cells without ESS65-Cl and IR-780). ****P* <0.001.


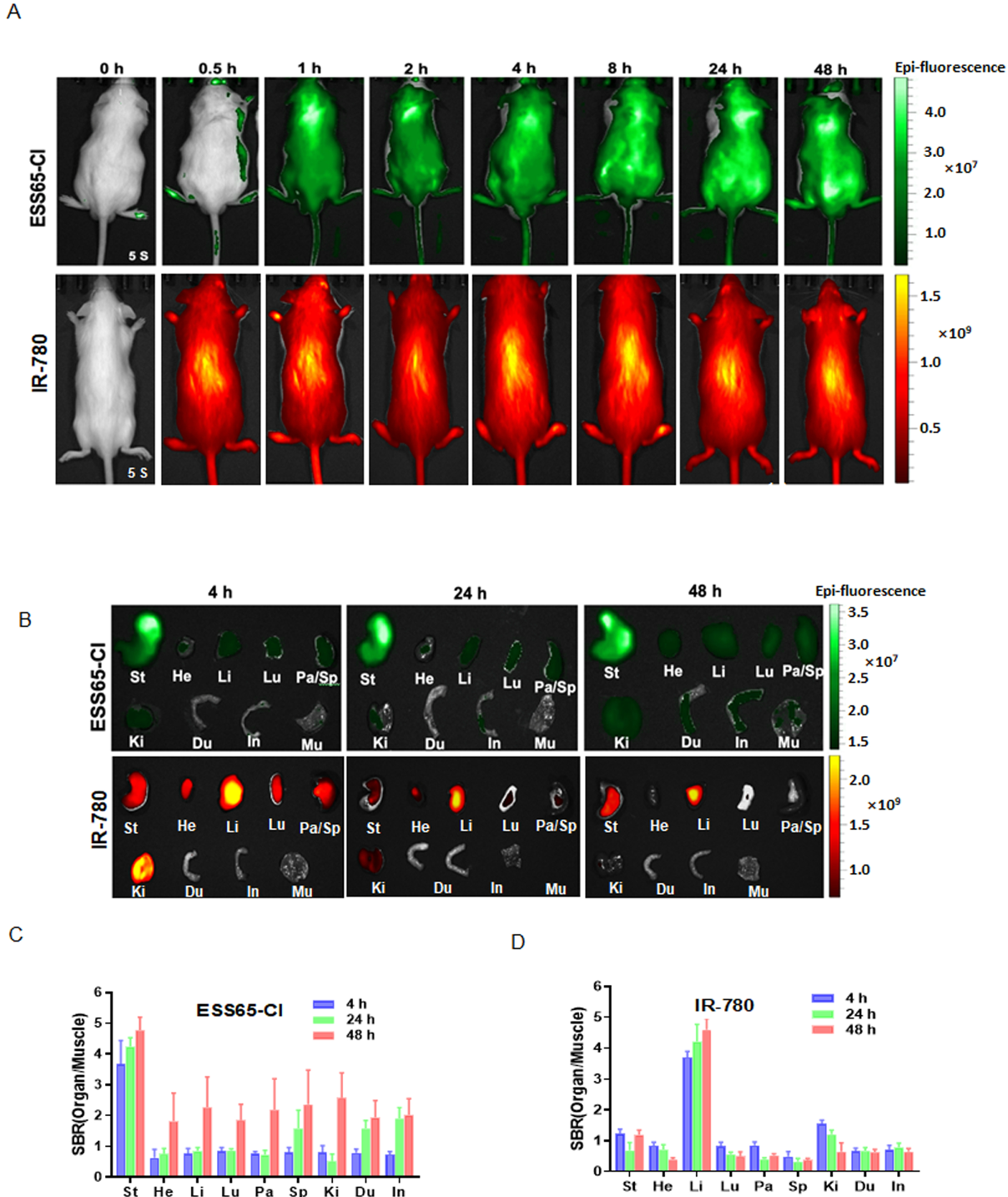


**Figure S3.** In vivo biodistribution and clearance of NIR fluorophores ESS65-Cl and IR-780 in mice. 200 nm of ESS65-Cl (100 µL, 700 nm channel) or 100 nm of IR-780 (100 µL, 800 nm channel) were injected intravenously into CD-1 mice, and their NIR fluorescence signals were observed at 0.5, 1, 2, 4, 8, 24, and 48 h post-injection. (A) Real-time NIR fluorescence imaging of mice at different times. (B) NIR imaging of resected tissues and organs at 4, 24, and 48 h. (C and D) The SBR was calculated by comparing the signals of major organs against the surrounding muscle. Abbreviations used are Du, duodenum; He, Heart; In, intestine; Li, liver; Lu, lung; Mu, muscle; Pa, pancreas; Sp, spleen; St, stomach. Three mice were analyzed for each sample.


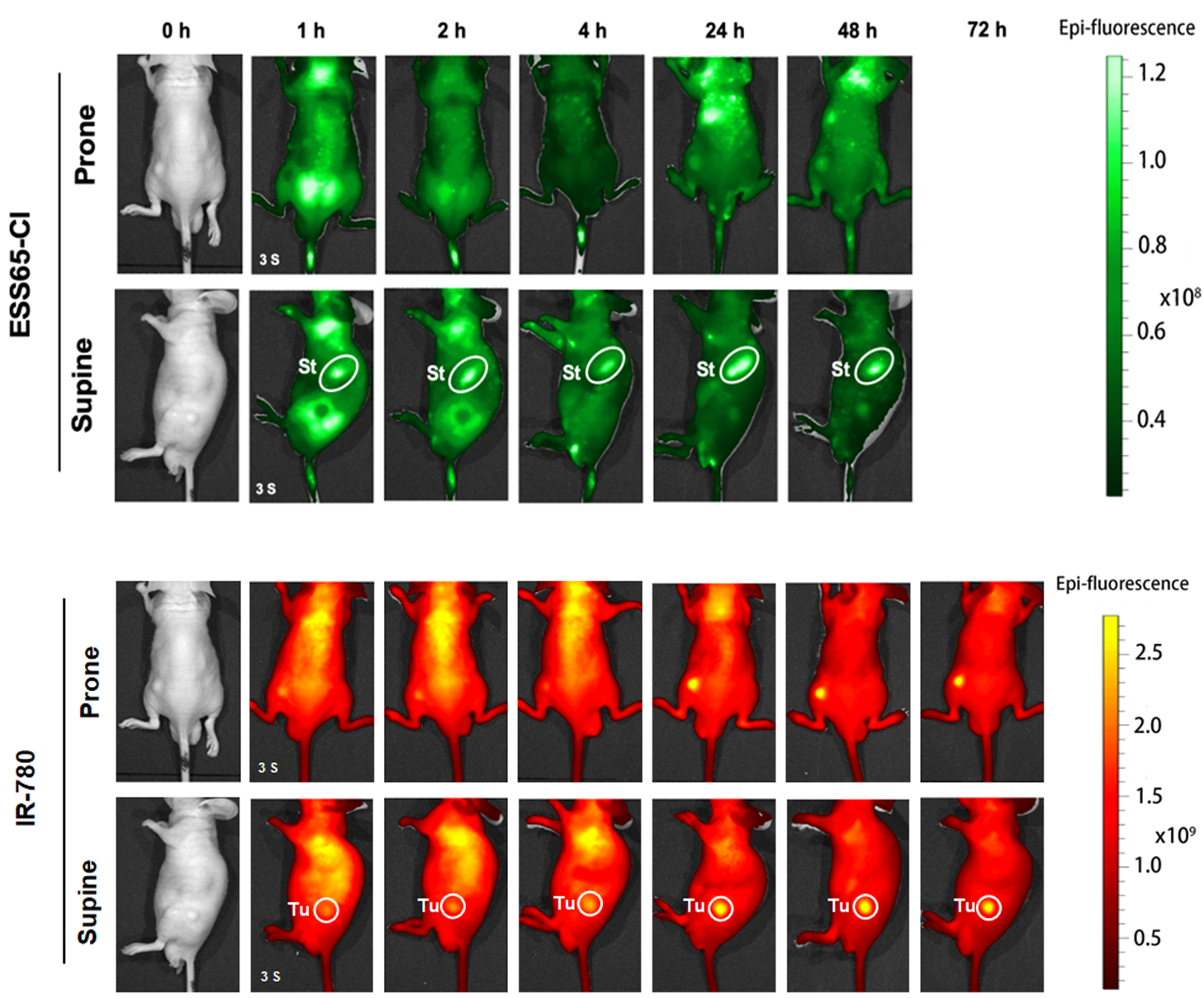


**Figure S4.** Real-time NIR targeted imaging after the injection of NIR fluorophores ESS65-Cl (λex = 641 nm; λem = 667 nm) and IR-780 (λex = 790 nm; λem = 805 nm) in the established mouse model of subcutaneous gastric tumor. 200 nm of ESS65-Cl (100 µL) was injected intravenously into gastric tumor-bearing mice 48 h prior to imaging, and their NIR fluorescence signals were observed at 1, 2, 4, 24, and 48 h post-injection. 100 nm of IR-780 (100 µL) was injected intravenously into gastric tumor-bearing mice 72 h prior to imaging, and their NIR fluorescence signals were observed at 1, 2, 4, 24, 48, and 72 h post-injection.


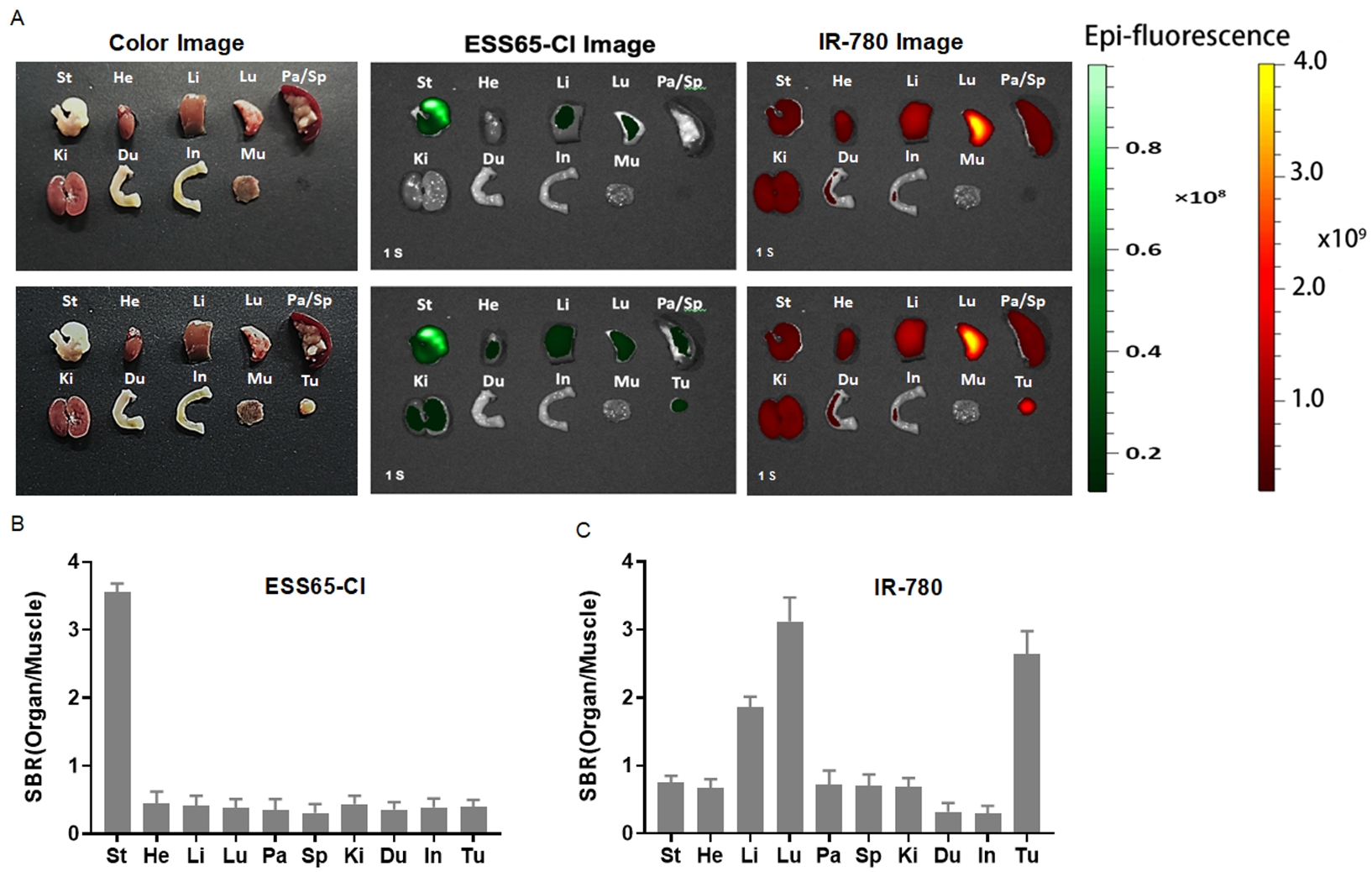


**Figure S5**. *In vivo* biodistribution and clearance of NIR fluorophores ESS65-Cl and IR-780in the established mouse model of subcutaneous gastric tumor. (A) NIR imaging of resected tissues and organs with ESS65-Cl (700 nm channel) and IR-780 (800 nm channel). (B,C) The SBR was calculated by comparing the signals of major organs against the surrounding muscle. Abbreviations used are Du, duodenum; He, heart; In, intestine; Li, liver; Lu, lung; Mu, muscle; Pa, pancreas; Sp, spleen; St, stomach; Tu, tumor. Three mice were analyzed for each sample.


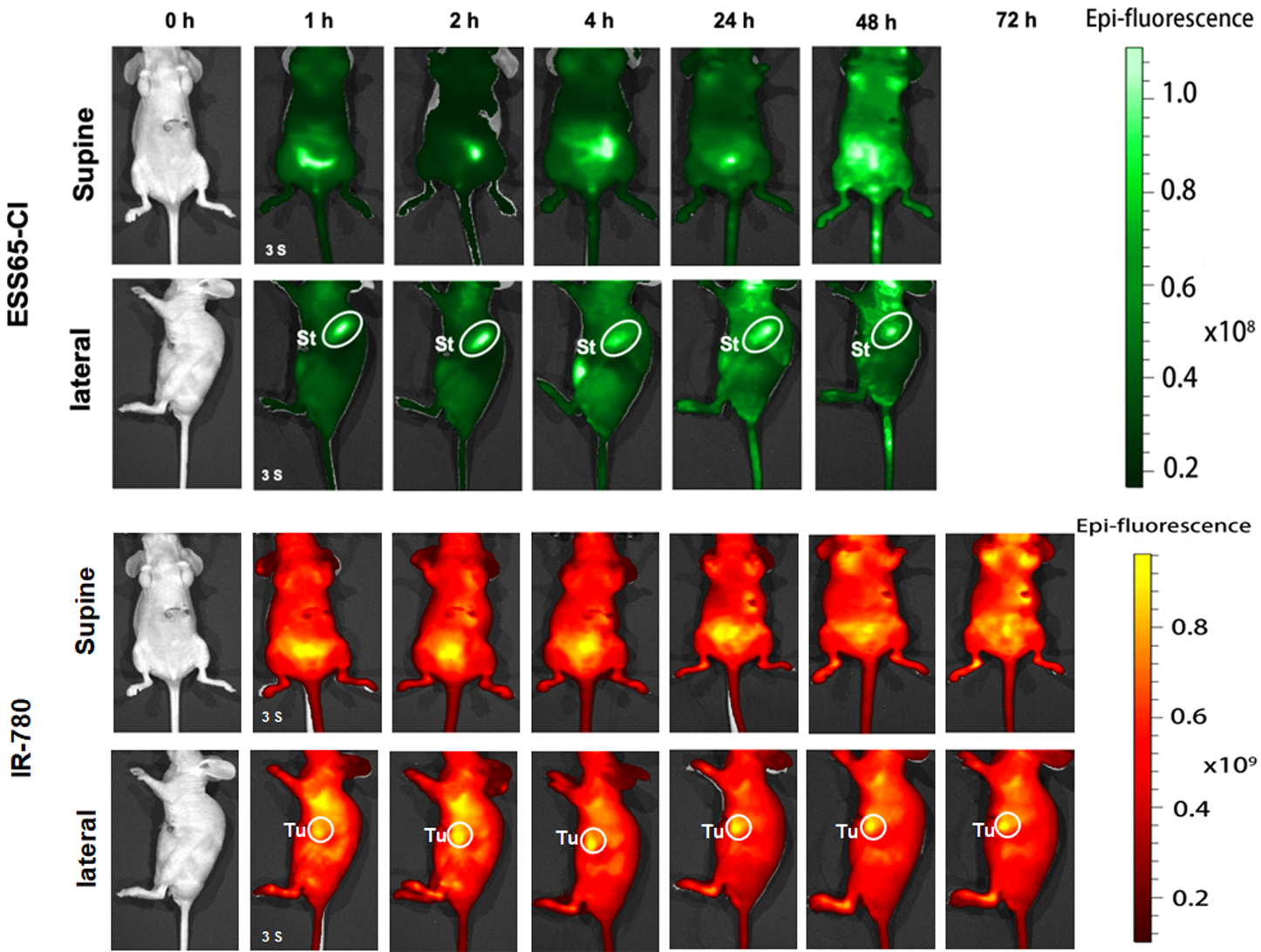


**Figure S6.** Real-time NIR targeted imaging after intravenous injections of NIR fluorophores ESS65-Cl (700 nm channel) and IR-780 (800 nm channel) in the established mouse model of orthotopic gastric tumor. 200 nm of ESS65-Cl (100 µL) was injected intravenously into orthotopic gastric tumor-bearing mice 48 h prior to imaging, and their NIR fluorescence signals were observed at 1, 2, 4, 24, and 48 h post-injection. 100 nm of IR-780 (100 µL) was injected intravenously into gastric tumor-bearing mice 72 h prior to imaging, and their NIR fluorescence signals were observed at 1, 2, 4, 24, 48, and 72 h post-injection.


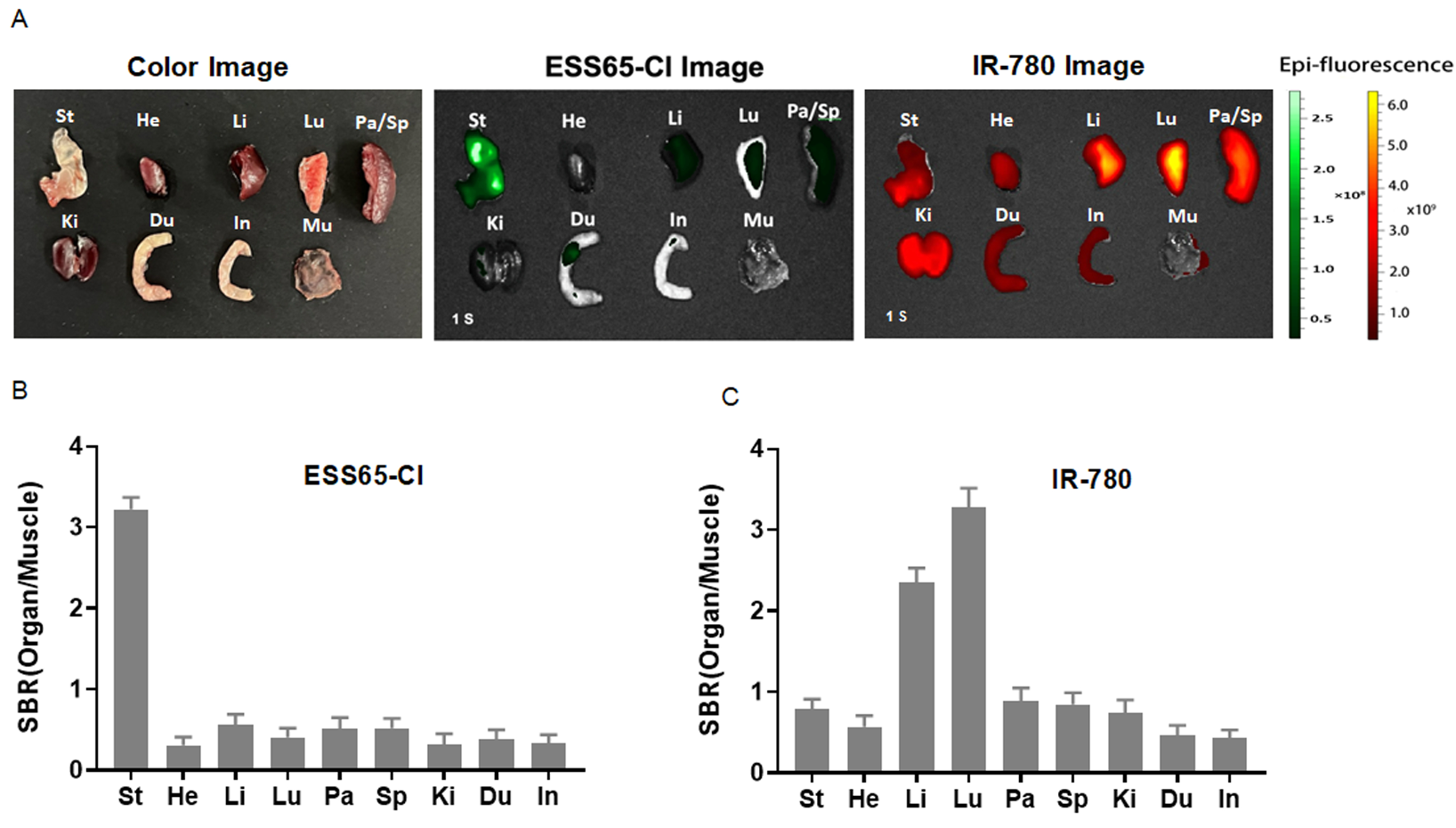


**Figure S7**. *In vivo* biodistribution and clearance of NIR fluorophores (ESS65-Cl and IR-780) in the established mouse model of orthotopic gastric tumor. (A) NIR imaging of resected tissues and organs with ESS65-Cl (700 nm channel) and IR-780 (800 nm channel). (B,C) The SBR was calculated by comparing the signals of major organs against the surrounding muscle. Abbreviations used are Du, duodenum; He, heart; In, intestine; Li, liver; Lu, lung; Mu, muscle; Pa, pancreas; Sp, spleen; St, stomach; Tu, tumor. Three mice were analyzed for each sample.
